# The moderating role of psychosocial working conditions on the long-term relationship between depressive symptoms and work ability among employees from the Baby Boom generation

**DOI:** 10.1007/s00420-020-01570-1

**Published:** 2020-09-08

**Authors:** Jeannette Weber, Hans Martin Hasselhorn, Daniela Borchart, Peter Angerer, Andreas Müller

**Affiliations:** 1 Institute of Occupational, Social and Environmental Medicine, Centre for Health and Society, Faculty of Medicine, Heinrich-Heine-University of Düsseldorf, Moorenstr. 5, 40225 Düsseldorf, Germany; 2 Department of Occupational Health Science, School of Mechanical Engineering and Safety Engineering, University of Wuppertal, Gaußstr. 20, 42119 Wuppertal, Germany; 3 Institute of Psychology, Work- and Organizational Psychology, University Duisburg-Essen, Universitätsstr. 2, 45141 Essen, Germany

**Keywords:** Mental health, Aging, lidA cohort study, Occupational disability, Workplace

## Abstract

**Objective:**

Mental disorders have been identified as a leading cause for reduced work ability in industrialized countries. Identification of workplace factors that can increase the work ability of employees with depressive symptoms from the Baby Boom generation is, therefore, highly relevant. This study thus aims to investigate whether changes in psychosocial working conditions can moderate the negative association between depressive symptoms and work ability.

**Methods:**

Two waves with a 3-year time lag of the German lidA cohort study with 3609 participants born in 1959 and 1965 (aged 46 and 52 years at first wave) were analyzed. Self-report data about depressive symptoms at baseline and changes of working conditions from baseline to follow-up were used to calculate main and interaction effects on perceived work ability at follow-up. These analyses were controlled for baseline work ability and working conditions.

**Results:**

Depressive symptoms were predictive for an unfavorable course of work ability from baseline to follow-up (*B* = − 0.173, 95% CI = − 0.219 to − 0.128). However, no interaction effect between depressive symptoms and psychosocial working conditions was found. Instead, independent from the level of depressive symptoms, a decrease in quantitative demands (*B* = − 0.279, 95% CI = − 0.326 to − 0.232) and increases in leadership quality (*B* = 0.242, 95% CI = 0.192–0.292) and development opportunities (*B* = 0.177, 95% CI = 0.127–0.277) were related to a more favorable course of work ability. Only small effects were found for social support (*B* = 0.057, 95% CI = 0.008–0.106) and job control (*B* = 0.043, 95% CI = − 0.005–0.091).

**Conclusions:**

The results indicate that the lagged and negative effect of depressive symptoms on work ability was not moderated by changes in psychosocial working conditions. However, the promotion of favorable working conditions may contribute to a positive development of work ability among employees from the Baby Boom generation independently from the level of depressive symptoms.

**Electronic supplementary material:**

The online version of this article (10.1007/s00420-020-01570-1) contains supplementary material, which is available to authorized users.

## Background

Mental disorders are the most common cause for occupational disability in industrialized countries (Leijten et al. 2015; Punakallio et al. 2014; Rehfeld 2006; Theis et al. 2018; Tuomi et al. 1991). Furthermore, the risk for occupational disability due to depression seems to increase with age (Ahola et al. 2011; Lagerveld et al. 2010). This relationship gains increasing importance due to the burden of demographic change and upcoming retirement of the Baby Boom generation on pension systems and labor markets. To reduce numbers of occupational disability and early retirement due to depressive symptoms, a thorough understanding which factors foster the work ability of employees with depressive symptoms from the Baby Boom generation is needed.

Good work ability can be achieved if personal resources (e.g. knowledge, skills and health) conform to psychosocial working conditions (Ilmarinen 2001; Kooij 2015). Depressive symptoms are related with loss of personal resources, for example including concentration difficulties or lack of energy (World Health Organization 2018). As a result, employees with depressive symptoms might particularly be dependent on additional environmental resources in terms of good working conditions to maintain their work ability.

Furthermore, psychosocial working conditions might differently affect work ability of employees with and without depressive symptoms. For example, healthy individuals might perceive some job demands as a challenge, which might positively affect mental well-being and work ability (Van den Broeck et al. 2010). Instead, individuals with depressive symptoms might experience challenging job demands as a burden due to fatigue, concentration difficulties, reduced working capacity and self-efficacy (Andersen et al. 2012). High job demands might, therefore, lead to the perception of decreased work ability among employees with depressive symptoms but not necessarily among healthy employees. Previous research on the interrelation between psychosocial working conditions, mental health and work ability has focused on overall working populations aged between 18 and 67 years. Those studies have not observed significant interactions between mental health and psychosocial working conditions and rather pointed to independent and additive effects on work ability (Hjarsbech et al. 2013; Leijon et al. 2017; Munir et al. 2011). To the best of our knowledge, similar research that specifically focused on employees of the Baby Boom generation is missing.

There are several reasons why psychosocial working conditions might have age-dependent moderating effects on the association between depressive symptoms and work ability. Work and life goals might change from knowledge attainment into emotional- and social related goals due to the perception of limited future time perspectives (also called socioemotional selectivity theory; Carstensen et al. (1999)). Consequently, also the role of working conditions might change during lifetime (Zacher and Schmitt 2016). For example, development opportunities might be most important for younger employees and increase their work ability irrespective of whether depressive symptoms are present or not (Carstensen et al. 1999; Leijon et al. 2017). With increasing age, social support might be more appreciated and development opportunities be valued to a smaller extent (Carstensen et al. 1999). Furthermore, self-efficacy for development seems to decrease with age (Maurer 2001). In combination with depressive symptoms, development opportunities might, therefore, be perceived as extra burden by older employees. Hence, the findings on younger working generations might not be valid for the Baby Boom generation consisting of middle-aged and older employees.

From a methodological perspective, previous research on interrelations between psychosocial working conditions, mental health and work ability has assessed working conditions only once at baseline (Hjarsbech et al. 2013; Leijon et al. 2017; Munir et al. 2011). However, with increasing age, employees may find it increasingly difficult to adapt to job changes due to resource loss and reduced reserve capacity (i.e. potential for maximum performance), which may lead to a perceived decline of individual job-fit (Baltes and Baltes 1990; Yeatts et al. 2000). It may, therefore, be advisable to consider changes of working conditions as well. In addition, differences in education, work tasks and expectations might lead to distinct effects of psychosocial working conditions depending on sex and occupational group (Bouville et al. 2018; Leijon et al. 2017), but stratification for those variables was not performed by previous research (Hjarsbech et al. 2013; Leijon et al. 2017; Munir et al. 2011). Furthermore, the possibility of reverse causation should be taken into account, because reduced work ability might increase the risk for depressive symptoms and lead to poorer appraisal of working conditions (Ford et al. 2014; Lee et al. 2017).

The aim of this study was, therefore, to expand previous research on modifying effects of working conditions on the relationship between depressive symptoms and work ability by examining a representative sample of a middle-aged subgroup from the Baby Boom generation in Germany and using a longitudinal design. In doing so, this study will not only investigate effects of working conditions at baseline but also specifically effects of changes in working conditions from baseline to follow-up by the use of change scores. Due to their association with the study variables, analyses are further controlled for year of birth, sex and physical activity (Campos-Serna et al. 2013; van den Berg et al. 2009; Wittchen et al. 2011). In addition, stratified analyses will be performed for occupational group in terms of unqualified, qualified and highly qualified work and for sex. Furthermore, analysis of reverse causation will be performed.

## Methods

### Study design and sample

Data of the first and second waves of the German lidA (German: "Leben in der Arbeit") cohort study was used. A detailed description of study design and sampling is given elsewhere (Hasselhorn et al. 2014; Schröder et al. 2013; Steinwede et al. 2015). In brief, the first wave (2011) comprises a random sample of 6585 participants born in 1959 (aged 52 years) or 1965 (aged 46 years) and is representative for the socially insured working population of same age. The sample excludes civil servants with the inaugurated status as “Beamter” (to be distinguished to civil servants working under private labor and collective bargaining laws) and all self-employed persons (Hasselhorn et al. 2014). Data were collected by computer-assisted interviews (CAPI). During the first wave, a response rate of 27.3% was achieved [RR5 according to standards of the American Association for Public Opinion Research (American Association for Public Opinion Research 2011)]. In 2014, 4244 participants could be interviewed again (Hasselhorn et al. 2014; Steinwede et al. 2015). Written informed consent for address storage and participation in the second wave was obtained from each of those participants. After each interview, participants were further asked to fill in a paper and pencil questionnaire covering the simplified version of Beck’s Depression Inventory (BDI-V). For this study, only respondents who were employed at both time points (*n* = 3961) and had returned the BDI-V questionnaire were included, leaving 3609 participants. Based on a previous meta-analysis about stressor-strain effects, a 3-year follow-up was assumed to be suitable to identify lagged effects of health and working conditions on work ability (Ford et al. 2014). Ethical approval for this study was given by the Ethics Committee of the University of Wuppertal in Germany (December 5th, 2008).

### Measures

Work ability was measured by the second dimension of the validated Work Ability Index [WAI (Hasselhorn and Freude 2007; Tuomi et al. 1999)]. Previous validation studies have suggested a two-factorial structure of the WAI with a health-related and a subjective work ability factor (Freyer et al. 2019; Martus et al. 2010). Since using the health-related factor might have introduced a construct overlap to depressive symptoms, the second dimension was used to measure subjective work ability. Participants rated their work ability in regard to physical and mental work demands on a 5-point rating scale from 1 = ‘very poor’ to 5 = ‘very good’. Furthermore, participants indicated whether they mainly performed physical, mental or likewise physical and mental work tasks. As recommended by the authors of the WAI (Hasselhorn and Freude 2007; Tuomi et al. 1999), ratings on work ability were weighted for indicated work tasks: the predominant task was weighted by 1.5, the subordinate by 0.5. No weighting was applied for likewise physical and mental tasks. Finally, both ratings were summed up to a score ranging from 2 to 10 with higher values corresponding to better work ability.

Depressive symptoms were measured by a simplified, applied and validated German version of the Beck's Depression Inventory (Rose et al. 2015; Schmitt et al. 2003). This version constitutes of 20 items whether participants experience depressive symptoms on a 6-point rating scale from 0 = ‘never’ to 5 =  ‘almost always’. Those ratings were summed up to a scale ranging from 0 to 100 with higher values corresponding to higher levels of depressive symptoms.

Quantitative job demands, job control, development opportunities, social support and quality of leadership were chosen for the analysis as previous research has identified those working conditions to be important risk factors or resources for work ability and sick leave in persons with depressive symptoms (White et al. 2013). Those variables were measured by a modified German version of the validated Copenhagen Psychosocial Questionnaire [COPSOQ (Nübling et al. 2011; Pejtersen et al. 2009)]. Scales consisted of three items each with a 5-point rating scale (individual scale items are listed in Online Resource (1). After transforming ratings on each item to a range from 0 to 100, mean scores were calculated per scale if at least two items were answered. Higher values correspond to higher levels of the respective working condition.

The analyses were controlled for sex, year of birth and physical activity (1 = '‘no physical activity’ to 4 = ‘several times per week intensive exercise’). Occupational group was measured in terms of unqualified (e.g. unskilled work, cashiers, waiters), qualified (e.g. nurses, hairdressers, administrative assistants) and highly qualified (e.g. managers, engineers, physicians) work following the validation study of the Blossfeld classification scheme (Blossfeld 1985; Wirth et al. 2009).

### Statistical analyses

In the final dataset 1.3% of values for work ability, working conditions and physical activity were missing, with highest proportions of missing values being observed for quality of leadership (wave 1: *n* = 124, 3.4%, wave 2: *n* = 143, 4.0%) and social support (wave 1: *n* = 159, 4.4%; wave 2: *n* = 183, 5.1%). For all other variables, the proportion of missing values was below 0.5%. Since imputation of missing values has been recommended over analysis of complete cases only (van der Heijden et al. 2006), missing values for work ability, working conditions and physical activity were imputed by expectation maximization. This method is deemed to be acceptable when less than 5% of the dataset is missing in case of missing at random (Scheffer 2002). To measure scale reliability, Cronbach’s alpha values were calculated. The reliability of change scores was calculated using the formula by Traub with values of > 0.5 demonstrating good reliability (Smith and Beaton 2008; Traub 1994).

The use of change scores has been recommended when the research focus is on the level of change and when those changes are assumed to result from actual changes in working conditions (Smith and Beaton 2008). Therefore, change scores of working conditions were computed (score of 2nd wave—score of 1st wave), with positive values indicating an increase and negative values indicating a decrease of job demands and resources. Prior to analysis, baseline and change scores for working conditions and depressive symptoms were z-standardized. Then two-way interaction terms between depressive symptoms and changes in working conditions were constructed. Multiple linear regression analyses were conducted using work ability from the second wave as the dependent variable and baseline levels of depressive symptoms and changes in working conditions as independent variables. Those analyses were controlled for sex, year of birth, physical activity, baseline work ability and baseline working conditions. Furthermore, analyses were repeated and stratified for either occupational group or sex. No substantial multicollinearity was observed since in all analyses tolerance (TOL) was always higher than 0.40 and the variance-inflating factor (VIF) never exceeded 2.1.

Reverse causation was tested by regressing the z-standardized values of work ability at wave 1 on working conditions or depressive symptoms at wave 2, controlling for all other variables at baseline.

Statistical significance was assumed at a p-level of < 0.05. Due to reduced power in interaction analyses also p-levels of < 0.10 are reported for interaction effects as trends (Aguinis 1995; Burks et al. 2019). All analyses were conducted with IBM SPSS Statistics 24.

## Results

### Descriptive results

Descriptive statistics for respondents participating at both waves and dropouts are displayed in Table 1. Respondents were more likely to have qualified or highly qualified work, reported more physical activity, higher work ability and less depressive symptoms. Respondents also reported higher levels of favorable working conditions except for quantitative demands, which were higher than in participants who were excluded.

**Table 1:** Drop-out analysis with study variables at wave 1, data without imputation

	Drop-outs (*n* = 2784)	Respondents (*n* = 3609)	Difference test
	%	%	*χ*^2^
Female	52.2	54.0	2.074
Year of birth			1.200
1965	56.8	55.4	
1959	43.2	44.6	
Occupational group			81.747*
Unqualified work	45.8	34.6	
Qualified work	37.9	45.1	
Highly qualified work	16.3	20.4	

	Mean (SD)	Mean (SD)	*t*
Physical activity	2.23 (0.82)	2.36 (0.80)	− 6.361*
Work ability	7.85 (1.65)	8.08 (1.50)	− 5.661*
Quantitative demands	43.30 (23.89)	45.27 (23.35)	− 3.282*
Decision authority	35.58 (27.94)	38.91 (27.50)	− 4.736*
Development	62.07 (24.93)	65.30 (23.12)	− 5.263*
Social support	63.87 (22.88)	65.47 (21.10)	− 2.808*
Leadership	53.47 (24.39)	54.81 (23.74)	− 2.142*
Depression	20.94 (14.69)^1^	19.76 (13.24)	2.983*

^1^Data of 1844 participants, who have filled out the BDI-V questionnaire at wave 1; drop-outs = participants who were only employed or participating at wave 1 or did not fill out BDI-V, respondents = participants who were participating and employed at both waves and filled out BDI-V, *n* = number of participants, SD = standard deviation, *χ*^2^ = Chi-squared test for differences between drop-outs and responder, *t* = *t*-test for independent samples for differences between drop-outs and respondents, level of significance (two-tailed): *p < 0.05

Descriptive statistics for respondents after imputation of missing data, correlation between study variables and scale reliability are given in Table 2. The Cronbach’s alpha values indicate that scale reliability for quantitative demands, development opportunities and quality of leadership was good, whereas the reliability for job control and social support was questionable.

**Table 2:** Study characteristics, correlations between study variables and scale reliability

	M (SD)	1	2	3	4	5	6	7	8	9	10	11	12	13
T1														
1. Work ability	8.08 (1.50)													
2. Quant. demands	45.26 (23.34)	− 0.195*	(0.806)											
3. Control	38.94 (27.48)	0.151*	0.055*	(0.659)										
4. Development	65.30 (23.11)	0.216*	0.108*	0.401*	(0.810)									
5. Social Support	65.44 (20.74)	0.130*	− 0.114*	0.127*	0.186*	(0.666)								
6. Leadership	54.91 (23.48)	0.239*	− 0.194*	0.207*	0.287*	0.246*	(0.819)							
7. Depression	19.76 (13.24)	− 0.363*	0.186*	− 0.126*	− 0.208*	− 0.186*	− 0.227*							
T2														
8. Work ability	7.92 (1.50)	0.501*	−0.116*	0.133*	0.157*	0.083*	0.160*	− 0.309*						
Change (T2-T1)														
9. Quant. Demands	− 0.26 (21.52)	0.064*	− 0.449*	− 0.008	0.002	0.043*	0.066*	− 0.038*	− 0.114*	[0.562]				
10. Control	0.69 (25.71)	0.001	− 0.001	− 0.467*	− 0.108*	− 0.022	− 0.059*	0.002	0.042*	− 0.020	[0.249]			
11. Development	− 2.49 (20.31)	− 0.060*	− 0.019	− 0.139*	− 0.495*	− 0.054	− 0.110*	0.031	0.056*	− 0.013	0.160*	[0.524]		
12. Social Support	− 1.33 (20.74)	− 0.040*	0.004	− 0.044*	− 0.076*	− 0.494*	− 0.059*	0.008	0.061*	− 0.065*	0.083*	0.117*	[0.397]	
13. Leadership	− 1.15 (23.74)	− 0.069*	0.089*	− 0.067*	− 0.091*	− 0.052*	− 0.467*	0.027	0.117*	− 0.154*	0.117*	0.197*	0.151*	[0.675]

*M* mean, *SD* standard deviation, *T1* wave 1, *T2* wave 2; Spearman correlation; in round brackets = Cronbach’s alpha, in square brackets = reliability score by Traub (1994); * *p* < 0.05; means, standard deviations and correlations are shown for data after imputation; reliability scores are shown for data before imputation

According to the recommended cut-off value of 35 for the BDI-V (Schmitt et al. 2006), 13.1% of all respondents had an increased risk for clinical relevant depression. The proportion of female participants with increased risk for depression was higher than the proportion of male participants (15.8% vs 9.9%; *χ*^2^(1) = 26.726, *p* < 0.05). Furthermore, differences in the proportion of employees with increased risks of depression between unqualified (13.4%), qualified (14.1%) and highly qualified (10.4%) work groups was observed (*χ*^2^(2) = 6.107, *p* < 0.05). Among all respondents, no difference between the level of depressive symptoms at wave 1 (*M* = 19.73, SD = 13.20) and 2 (*M* = 19.74, SD = 13.81) was observed (paired t-test: *t*(3301) = − 0.028, *p* = 0.98).

### Main effects of working conditions and depressive symptoms

To analyze relationships between working conditions, depressive symptoms and work ability, multiple linear regression analysis was applied and results are shown in Table 3 for the non-stratified sample and in Table 4 for the stratified sample.

**Table 3:** Multiple linear regression analysis predicting work ability at wave 2, data shown after imputation

	I: main effects		II: main and interaction effects	
	*B*	95% CI	*B*	95% CI
T1 work ability	0.402*	0.372; 0.432	0.402*	0.372; 0.432
Sex (Ref. female)	0.032	− 0.050; 0.115	0.033	-0.050; 0.115
Year of birth (Ref. 1959)	0.026*	0.012; 0.039	0.025*	0.012; 0.039
Physical activity	0.105*	0.054; 0.156	0.105*	0.054; 0.156
Working conditions				
T1 quantitative demands	− 0.177*	− 0.227; − 0.127	− 0.177*	− 0.227; − 0.127
T1 control	0.058*	0.006; 0.110	0.058*	0.006; 0.110
T1 development	0.151*	0.095; 0.207	0.151*	0.095; 0.207
T1 social Support	0.003	− 0.048; 0.053	0.003	-0.048; 0.054
T1 leadership	0.142*	0.089; 0.195	0.141*	0.088; 0.194
Symptoms				
T1 depression	− 0.173*	− 0.219; -0.128	–0.175*	− 0.221; -0.129
Change in working conditions				
Δ quantitative demands	− 0.279*	− 0.326; -0.232	–0.280*	− 0.327; − 0.233
Δ control	0.043	− 0.005; 0.091	0.042	− 0.006; 0.090
Δ development	0.177*	0.127; 0.227	0.177*	0.127; 0.228
Δ social support	0.057*	0.008; 0.106	0.057*	0.008; 0.106
Δ leadership	0.242*	0.192; 0.292	0.242*	0.192; 0.292
Interactions				
T1 depression * Δ Quantitative demands			− 0.020	− 0.059; 0.020
T1 depression * Δ control			− 0.007	− 0.049; 0.035
T1 depression * Δ Development			0.001	− 0.037; 0.039
T1 depression * Δ social Support			− 0.007	− 0.047; 0.032
T1 depression * Δ Leadership			− 0.001	− 0.042; 0.040
Model fit: *R*^2^	*F*(15,3593) = 126.003*, *R*^2^ = .345		*F*(20,3588) = 94.457*, *R*^2^ = .345	

*T1 *wave 1, Δ change from wave 1 to wave 2 with positive values representing an increase and negative values representing a decrease in working condition, *B *unstandardized regression coefficient, *CI* confidence interval, *Ref.* reference category; Levels of significance (two-tailed): ^#^*p* < 0.10, *p < 0.05

**Table 4:** Multiple linear regression analysis predicting work ability at wave 2, stratified for sex occupational group, data shown after imputation

	Stratified for sex				Stratified for occupational group					
	Female, *n* = 1949		Male, *n* = 1660		Unqualified work, *n* = 1236		Qualified work, *n* = 1613		Highly qualified work, *n* = 728	
	*B*	95% CI	*B*	95% CI	*B*	95% CI	*B*	95% CI	*B*	95% CI
T1 Work ability	0.392*	0.349; 0.434	0.406*	0.364; 0.449	0.411*	0.360; 0.461	0.367*	0.322; 0.415	0.425*	0.356; 0.493
Year of birth (Ref. 1959)	0.029*	0.010; 0.048	0.021*	0.002; 0.040	0.023	0.000; 0.047	0.033*	0.013; 0.054	0.013	− 0.016; 0.043
Physical activity	0.127*	0.052; 0.202	0.076*	0.007; 0.145	0.087	− 0.001; 0.175	0.129*	0.050; 0.207	0.035	− 0.072; 0.143
Sex (Ref. female)					− 0.037	− 0.187; 0.113	0.072	− 0.070; 0.215	0.258*	0.067; 0.450
Working conditions										
T1 Quantitative demands	− 0.195*	− 0.264; − 0.126	− 0.152*	− 0.223; − 0.080	− 0.179*	− 0.269; − 0.090	− 0.217*	− 0.291; − 0.143	− 0.200*	− 0.381; − 0.081
T1 Control	0.069	− 0.004; 0.142	0.030	− 0.045; 0.104	0.019	-0.073; 0.111	0.103*	0.026; 0.180	− 0.004	− 0.120; 0.112
T1 Development	0.076*	0.001; 0.152	0.275*	0.190; 0.360	0.126*	0.036; 0.216	0.105*	0.010; 0.200	0.126	− 0.020; 0.272
T1 Social Support	− 0.006	− 0.079; 0.066	0.020	− 0.052; 0.091	0.018	− 0.067; 0.102	− 0.027	− 0.104; 0.051	0.115	− 0.005; 0.235
T1 Leadership	0.182*	0.108; 0.256	0.088*	0.012; 0.164	0.101*	0.010; 0.191	0.161*	0.080; 0.242	0.123*	0.007; 0.239
Symptoms										
T1 Depression	− 0.179*	− 0.241; -0.117	− 0.162*	− 0.231; -0.093	− 0.258*	− 0.335; − 0.182	− 0.142*	− 0.212; -0.073	− 0.133*	− 0.242; − 0.024
Change in working conditions										
Δ Quantitative demands	− 0.286*	− 0.352; − 0.220	− 0.267*	− 0.335 − 0.199	− 0.255*	− 0.333; -0.176	− 0.320*	− 0.395; − 0.245	− 0.304*	− 0.407; − 0.201
Δ Control	0.059	− 0.006; 0.125	0.032	− 0.040; 0.105	− 0.043	− 0.119; 0.034	0.136*	0.060; 0.213	− 0.002	− 0.114; 0.111
Δ Development	0.144*	0.077; 0.211	0.230*	0.153; 0.308	0.159*	0.081; 0.238	0.171*	0.090; 0.253	0.202*	0.079; 0.326
Δ Social Support	0.023	− 0.048; 0.093	0.105*	0.036; 0.175	0.110*	0.029; 0.192	− 0.013	− 0.087; 0.062	0.122*	0.008; 0.236
Δ Leadership	0.295*	0.226; 0.363	0.157*	0.083; 0.232	0.183*	0.098; 0.267	0.255*	0.180; 0.330	0.298*	0.179; 0.416
Interactions										
T1 Dep. * Δ Quantitative demands	− 0.030	− 0.085; 0.025	− 0.007	− 0.065; 0.051	− 0.012	− 0.072; 0.048	− 0.011	− 0.077; 0.055	0.048	− 0.140; 0.044
T1 Dep. * Δ Control	− 0.048#	− 0.102; 0.006	0.063#	− 0.005;0.131	− 0.024	− 0.085; 0.037	0.022	− 0.045; 0.089	0.004	− 0.113; 0.120
T1 Dep. * Δ Development	0.010	− 0.039; 0.059	− 0.019	− 0.084; 0.047	0.046#	− 0.009; 0.100	− 0.031	− 0.098; 0.035	− 0.024	− 0.133; 0.086
T1 Dep. * Δ Social Support	− 0.022	− 0.076; 0.032	0.029	− 0.031; 0.090	− 0.008	− 0.071; 0.056	− 0.010	− 0.071; 0.050	0.026	− 0.073; 0.124
T1 Dep. * Δ Leadership	0.008	− 0.047; 0.063	− 0.035	− 0.100; 0.029	− 0.014	− 0.078; 0.049	0.027	− 0.036; 0.089	− 0.026	− 0.144; 0.044
Model fit: *R*^2^	*F*(19,1929) = 51.443*, *R*^2^ = .336		*F*(19,1640) = 49.777*, *R*^2^ = .366		*F*(20,1215) = 33.137*, *R*^2^ = .353		*F*(20,1592) = 40.347*, *R*^2^ = .336		*F*(20,707) = 20.355*, *R*^2^ = .365	

*T1  *wave 1, *Δ* change from wave 1 to wave 2 with positive values representing an increase and negative values representing a decrease in working condition, *n*  number of participants, *B*  unstandardized regression coefficient, *CI* confidence interval, *Ref.* reference category; Levels of significance (two-tailed): * *p* < 0.05

Within the non-stratified sample and among all sub-groups, depressive symptoms and quantitative demands were negatively associated, whereas development opportunities and leadership were positively associated with a more favorable course of work ability. However, work ability was more strongly related to development opportunities in men and more strongly related to leadership quality in women. Small positive associations between work ability and job control and social support were found. Job control was only associated with work ability among persons in qualified work and social support was only associated with work ability in the male, unqualified and highly qualified sub-groups.

### Interaction effects of working conditions and depressive symptoms

No interactions between changes in working conditions and depressive symptoms were identified within the non-stratified sample (Table 3).

**Fig. 1:**
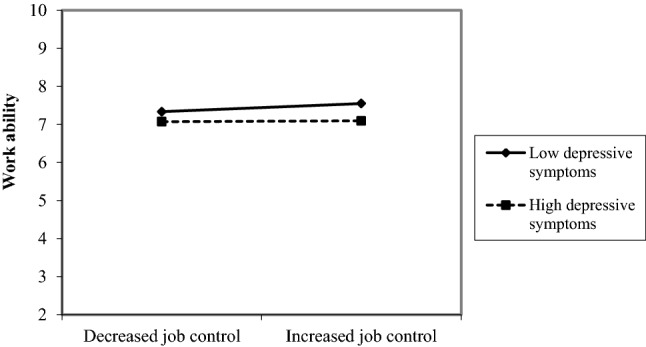
Interaction effect between depressive symptoms and change in job control in female participants

**Fig. 2:**
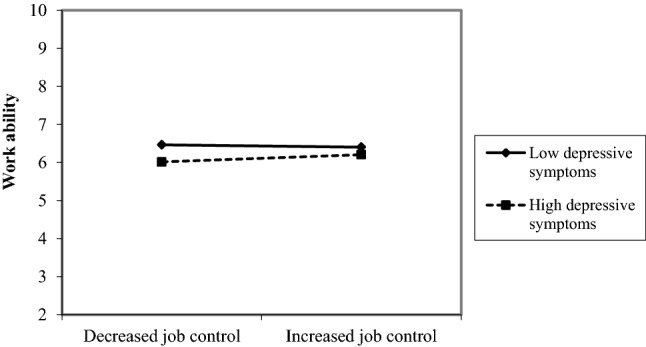
Interaction effect between depressive symptoms and change in job control in male participants

However, a trend of opposing interactions between depressive symptoms and change in job control indicate that in women an increase in job control was only associated with a more favorable course of work ability when depression levels were low (Fig. 1). In men, an increase in job control was only associated with a more favorable course of work ability when depression levels were high (Fig. 2).

**Fig. 3:**
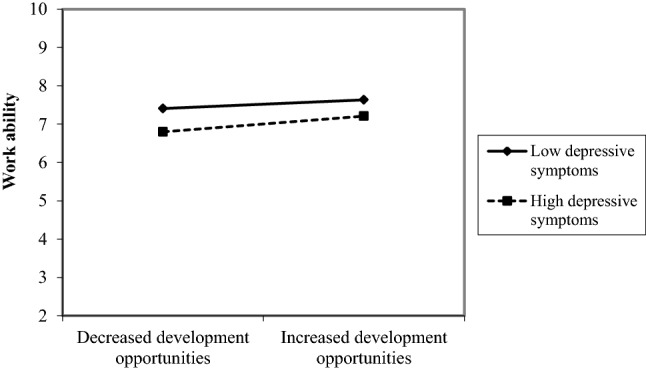
Interaction effect between depressive symptoms and change in development opportunities in participants with unqualified work

In addition, a trend of an interaction emerged between changes in development opportunities and depressive symptoms in participants with unqualified work: an increase in development opportunities was more strongly related to a more favorable course of work ability in persons with high compared to persons with low levels of depressive symptoms (Fig. 3).

### Reverse causation

Work ability at baseline was associated with future job control (*B* = 1.146, 95% CI = 0.329 to 1.963) and depressive symptoms (*B* = − 0.884, 95% CI = − 1.252 to − 0.517). Effects of reverse causation were insignificant for quality of leadership (*B* = 0.191, 95% CI = − 0.557 to 0.938), development opportunities (*B* = 0.469, 95% CI = − 0.167 to 1.104), quantitative demands (*B* = − 0.529, 95% CI = − 1.220 to 0.161) and social support (*B* = − 0.327, 95% CI = − 0.974 to 0.320).

## Discussion

This study analyzed the interplay between depressive symptoms and changes in working conditions on work ability in a representative sample of middle-aged employees from the Baby Boom generation in Germany. To summarize, depressive symptoms and changes in working conditions were predictive for the course of work ability from baseline to follow-up. However, no interaction effects between depressive symptoms and changes in working conditions were observed in the non-stratified sample. In accordance with similar previous research (Hjarsbech et al. 2013; Leijon et al. 2017; Munir et al. 2011), those results suggest that low levels of depressive symptoms at baseline, increasing levels of job resources and decreasing levels of job demands independently and additively contribute to higher work ability.

In light of previous evidence that depressive disorders often follow a chronic and recurrent course, the finding of a lagged effect of depressive symptoms on work ability after 3 years is not surprising and hints to an increasing negative effect due to a cumulative exposure to depression (Andrews 2001; Monroe and Harkness 2011). Interpreting our results in light of conventional effect sizes for small, medium and large effects (e.g. Cohen’s d) might be misleading due to the use of autoregressive models as outlined by Adachi and Willoughby (2015). By controlling for baseline work ability, variance in work ability at follow-up is reduced by the stability of work ability over time and by the bivariate correlation between predictors and work ability at baseline. Within this study, the stability of work ability and the bivariate correlation at baseline between depressive symptoms and working conditions had medium effect sizes. Therefore, the lagged effect of depressive symptoms and their interactions with working conditions might be more meaningful as conventional effect sizes would suggest. Our finding that even after a time-lag of 3 years, depressive symptoms are weakly associated with a decrease of work ability might indicate a need for interventions to maintain work ability of depressed middle-aged individuals. The facts that the 12-month prevalence rate of major depressive episodes in the adult population reaches 5.5% in high-income countries and that the risk for occupational disability due to depressive symptoms increases with age (Bromet et al. 2011; Lagerveld et al. 2010) emphasize this need.

One starting point may involve adaptation of psychosocial working conditions (Bonde 2008; Gragnano et al. 2018) so that we proposed that working conditions might moderate the effect of depressive symptoms on work ability among employees from the Baby Boom generation. However, we did not observe interaction effects between depressive symptoms and working conditions within the non-stratified sample. Our results rather point to independent effects of working conditions and depressive symptoms on work ability. In other words: the working conditions predicting work ability of the Baby Boom generation seem to be the same for depressed and non-depressed individuals. This result is consistent with earlier research on study populations with a wider range of age (Hjarsbech et al. 2013; Leijon et al. 2017; Munir et al. 2011). Nevertheless, the results do not exclude the possibility that other or more specific working conditions might moderate the relationship between depressive symptoms and work ability. As mentioned before, job demands might be classified as job challenges or job hindrances (Van den Broeck et al. 2010). Whereas healthy individuals might perceive one job demand as a challenge, individuals with depressive symptoms might perceive the same job demand as a burden. Therefore, the use of other more differentiated scales for psychosocial working conditions might yield different results. Furthermore, workplace factors such as stigmatization of depressive disorders (Thisted et al. 2018) or workplace bullying (Gragnano et al. 2018) might be other more important determinants of the perception of work ability among employees with depressive symptoms.

In contrast to the non-stratified sample, an interaction emerged among participants with unqualified work, indicating that increases in development opportunities are more strongly related to a favorable course of work ability when depressive symptoms are high. Furthermore, an increase in job control was positively related to the work ability of female participants with low depressive symptoms and of male participants with high depressive symptoms. So far, we have no theoretical explanation for those findings. Furthermore, the interactions are difficult to interpret, as gender is confounded with qualification in this study. Within the lidA cohort, female participants were more often pursuing qualified work than male participants were (Hasselhorn et al. 2016) and our findings suggest that workers need to be qualified to benefit from increased job control. Therefore, female non-depressed employees might have benefited from job control because of their qualification and not because they are female. Furthermore, low levels of job control might increase the risk for depressive symptoms (Theorell et al. 2015), but among older employees such associations were only found for less-educated employees (Mäcken 2019). Since male participants were more often pursuing unqualified work (Hasselhorn et al. 2016), male participants with high depressive symptoms at baseline might have been more likely than female participants to experience a decline of depressive symptoms and reported better work ability at follow-up if their job control increased during the course of our study. However, generally those interaction effects were rather small and did not attain statistical significance below a *p*-value of 0.05. In addition, multiple testing might have increased the chance for type I errors. Observed interaction effects within the stratified analyses should, therefore, only be considered as trends and need further verification.

Although our study provides only little indications that employees with depressive symptoms from the Baby Boom generation particularly benefit from an adaptation of working conditions, there were considerable main effects of changes of working conditions on work ability. Especially a decrease in quantitative demands, followed by an increase in quality of leadership and development opportunities was associated with a favorable course of work ability. Those results are in accordance with the job demands-resources model: high job demands (e.g. quantitative demands) and low resources (e.g. leadership quality, development opportunities) at work result in unfavorable work outcomes (Bakker and Demerouti 2007). Our stratified analyses further suggest that development opportunities are more strongly related to work ability in male employees, whereas leadership quality is more strongly related to work ability in female employees. Differences in work and social roles (Campos-Serna et al. 2013), qualification (Hasselhorn et al. 2016) and working life-courses (Engels et al. 2019) between men and women might be underlying causes for such variations.

Although the small and insignificant effects of job control and social support are unexpected, also other studies have only found limited evidence for an association between social support and work ability among employees aged 45 years and above (Koolhaas et al. 2014; McGonagle et al. 2015). One explanation might involve the formulation of scale items for social support: participants were asked in how far they received support by their colleagues but not whether this support was also needed. Furthermore, our stratified analyses suggest that effects of social support and job control depend on occupational group and sex. However, internal reliability was low for both scales and we recommend interpreting our results regarding job control and social support with caution.

## Strengths and limitations

By using a longitudinal approach, we were able to analyze changes in psychosocial working conditions over time and test the possibility of reverse causation. Reverse relationships between work ability and most working conditions were not observed, but our analyses suggest that reduced work ability may lead to depressive symptoms. This reverse causation effect should have been adjusted for by the longitudinal design with depressive symptoms predicting future work ability controlled for prior work ability. However, the use of only two time points may still lead to partly cross-sectional effects when analyzing change scores. Therefore, a longitudinal design with more than two time points may still be desirable (Taris and Kompier 2003) but may then raise concerns regarding the right time lag to potentially detect associations between changes in working conditions and work ability (de Lange et al. 2003).

A further strength of this study involves the representative study sample of two age groups of the “baby boom” generation, which represents a large part of the German workforce (Hasselhorn et al. 2014). A previous investigation confirmed that only minor differences in socioeconomic characteristics occurred between this study sample and all employees of those age groups who are liable to the German social security system (Schröder et al. 2013). The response rate of 27% during baseline data collection is, therefore, deemed sufficient (Hasselhorn et al. 2014). Nevertheless, differences between participants of both waves and those that only participated during the first wave raise concerns regarding a healthy worker effect. However, differences were rather small and due to the large sample size also minor variations may turn out to be statistically significant. In addition, inclusion of two comparatively young age groups of the Baby Boom generation limits representativeness to employees in the sixth decade. Even though perceived and actual age-related loss of some resources that are supposed to be important components of work ability starts from 30 to 40 years of age onwards [e.g. muscle mass (Heckhausen et al. 1989; JafariNasabian et al. 2017), fluid intelligence (Salthouse 2009)], decline of other resources (e.g. executive cognitive functioning) starts only later in life (Tombaugh et al. 1999). Therefore, different results regarding interactions between depressive symptoms and working conditions might be expected among employees with more advanced age.

As mentioned above, internal reliability for the job control and social support scales was low. Furthermore, especially change scores can suffer from low reliability increasing the risk for regression to the mean [RTM, i.e. extremely high values are more likely to decrease and extremely low values are more likely to increase at second measurement (Smith and Beaton 2008)]. Low reliability and RTM effects might have, therefore, obscured and reduced potential relationships between working conditions and work ability. However, reliability of change scores for quantitative demands, development opportunities and leadership quality was good. In addition, controlling for baseline variables should have adjusted potential RTM effects.

## Implications

Our results imply that changes in psychosocial working conditions might have similar effects on work ability of employees with and without depressive symptoms. Therefore, results of previous studies on the effectiveness of organizational-level interventions to promote health and well-being by adapting psychosocial working conditions (Bond and Bunce 2001; Bourbonnais et al. 2011; Montano et al. 2014; Müller 2016) might be generalizable to employees with depressive symptoms. Furthermore, the results of our study imply that quantitative job demands, development opportunities and leadership quality might offer better opportunities to enhance the work ability of employees from the Baby Boom generation than changing social support or job control. Last but not least, our stratified analyses imply that it might be useful to consider gender and type of occupation in the development of such organizational-level interventions to promote work ability of the Baby Boom generation. Nevertheless, future research might first try to extend our study results with a more differentiated distinction of occupational groups.

## Concluding remarks

This longitudinal study indicates that the lagged and negative effect of depressive symptoms on work ability was not moderated by changes in psychosocial working conditions in a sample of employees from the Baby Boom generation in Germany. Instead, promotion of positive psychosocial working conditions may contribute to a more favorable course of work ability irrespective whether depressive symptoms are present or not.

## Electronic supplementary material

Below is the link to the electronic supplementary material.

Additional file1 (PDF 496 kb)

Additional file2 (PDF 566 kb)

Additional file3 (PDF 561 kb)

Additional file4 (PDF 561 kb)

Additional file5 (PDF 580 kb)

Additional file6 (PDF 561 kb)
