# Supplementary material for: The moderating role of psychosocial working conditions on the long-term relationship between depressive symptoms and work ability among employees from the Baby Boom generation
Source: Int Arch Occup Environ Health. 2020 Sep 8;94(2):295–307. doi: 10.1007/s00420-020-01570-1 (PMC7872994; doi:10.1007/s00420-020-01570-1)
Supplement: Supplementary file 1 — Additional file1 (PDF 496 kb) [file 420_2020_1570_MOESM1_ESM.pdf]

Article title: The moderating role of psychosocial working conditions on the long-term relationship between depressive symptoms and work ability among employees from the Baby Boom generation
Journal name: International Archives of Occupational and Environmental Health
Author names: Jeannette Weber, Hans Martin Hasselhorn, Daniela Borchart, Peter Angerer, Andreas Müller
Corresponding author: Jeannette Weber, Institute of Occupational, Social and Environmental Medicine, Centre for Health and Society, Heinrich-Heine-University of Düsseldorf, Düsseldorf, Germany (email: jeannette.weber@uni-duesseldorf.de)

Online Resource 1

Scale items of the Copenhagen Psychosocial Questionnaire (COPSOQ) that were used in the lidA cohort study, cited from Pejtersen et al. 2009

Quantitative job demands: five point rating scale from 1 = always to 5 = never/hardly ever

1. Is your workload unevenly distributed so it piles up?
2. How often do you not have time to complete all your work tasks?
3. Do you get behind with your work?

Job control: five point rating scale from 1 = always to 5 = never/ hardly ever

1. Do you have a say in choosing who you work with?
2. Can you influence the amount of work assigned to you?
3. Do you have any influence on what you do at work?

Development opportunities: five point rating scale from 1 = to a very large extent to 5 = to a very small extent

1. Do you have the possibility of learning new things through your work?
2. Can you use your skills or expertise in your work?
3. Does your work give you the opportunity to develop your skills?

Social support: five point rating scale from 1 = always to 5 = never/ hardly ever

1. How often do you get help and support from your colleagues?
2. How often are your colleagues willing to listen to your problems at work?
3. How often do you talk with your colleagues about how well you carry out your work?

Quality of leadership: five point rating scale from 1 = to a very large extent to 5 = to a very small extent

1. To what extent would you say that your immediate superior makes sure that the individual member of staff has good development opportunities?

2. To what extent would you say that your immediate superior gives high priority to job satisfaction?
3. To what extent would you say that your immediate superior is good at solving conflicts?

Ratings on all items were reversed and transformed to a scale from 0 to 100.

Reference:

Pejtersen JH, Kristensen TS, Borg V, Bjorner JB (2009) The second version of the Copenhagen Psychosocial Questionnaire. Scand J Public Health 38:8-24 doi:10.1177/1403494809349858
