# Supplementary material for: The moderating role of psychosocial working conditions on the long-term relationship between depressive symptoms and work ability among employees from the Baby Boom generation
Source: Int Arch Occup Environ Health. 2020 Sep 8;94(2):295–307. doi: 10.1007/s00420-020-01570-1 (PMC7872994; doi:10.1007/s00420-020-01570-1)
Supplement: Supplementary file 2 — Additional file2 (PDF 566 kb) [file 420_2020_1570_MOESM2_ESM.pdf]

Article title: The moderating role of psychosocial working conditions on the long-term relationship between depressive symptoms and work ability among employees from the Baby Boom generation

Journal name: International Archives of Occupational and Environmental Health

Author names: Jeannette Weber, Hans Martin Hasselhorn, Daniela Borchart, Peter Angerer, Andreas Müller

Corresponding author: Jeannette Weber, Institute of Occupational, Social and Environmental Medicine, Centre for Health and Society, Heinrich-Heine-University of Düsseldorf, Düsseldorf, Germany (email: jeannette.weber@uni-duesseldorf.de)

Online Resource 2

Multiple linear regression analysis predicting work ability at wave 2, stratified for sex, data shown after imputation

	Female, n=1949		Male, n=1660	
	B	95% CI	B	95% CI
T1 Work ability	.392*	0.349; 0.434	.406*	0.364; 0.449
Year of birth (Ref. 1959)	0.029*	0.010; 0.048	0.021*	0.002; 0.040
Physical activity	0.127*	0.052; 0.202	0.076*	0.007; 0.145
Working conditions				
T1 Quantitative demands	-0.195*	-0.264; -0.126	-0.152*	-0.223; -0.080
T1 Control	0.069	-0.004; 0.142	0.030	-0.045; 0.104
T1 Development	0.076*	0.001; 0.152	0.275*	0.190; 0.360
T1 Social Support	-0.006	-0.079; 0.066	0.020	-0.052; 0.091
T1 Leadership	0.182*	0.108; 0.256	0.088*	0.012; 0.164
Symptoms				
T1 Depression	-0.179*	-0.241; -0.117	-0.162	-0.231; -0.093
Change in working conditions				
Δ Quantitative demands	-0.286*	-0.352; -0.220	-0.267*	-0.335 -0.199
Δ Control	0.059	-0.006; 0.125	0.032	-0.040; 0.105
Δ Development	0.144*	0.077; 0.211	0.230*	0.153; 0.308
Δ Social Support	0.023	-0.048; 0.093	0.105*	0.036; 0.175
Δ Leadership	0.295*	0.226; 0.363	0.157*	0.083; 0.232
Interactions				
T1 Dep. * Δ Quantitative demands	-0.030	-0.085; 0.025	-0.007	-0.065; 0.051
T1 Dep. * Δ Control	-0.048#	-0.102; 0.006	0.063#	-0.005;0.131
T1 Dep. * Δ Development	0.010	-0.039; 0.059	-0.019	-0.084; 0.047
T1 Dep. * Δ Social Support	-0.022	-0.076; 0.032	0.029	-0.031; 0.090
T1 Dep. * Δ Leadership	0.008	-0.047; 0.063	-0.035	-0.100; 0.029
Model fit: R^2	$F(19,1929)=51.443^*$, $R^2=.336$		$F(19,1640)=49.777^*$,	

Note: T1 = wave 1, Δ = change from wave 1 to wave 2 with positive values representing an increase and negative values representing a decrease in working condition, n = number of participants, B = unstandardized regression coefficient, CI = confidence interval, Ref. = reference category; Levels of significance (two-tailed): # p < .10, * $p < 0.05$
