# Supplementary material for: The moderating role of psychosocial working conditions on the long-term relationship between depressive symptoms and work ability among employees from the Baby Boom generation
Source: Int Arch Occup Environ Health. 2020 Sep 8;94(2):295–307. doi: 10.1007/s00420-020-01570-1 (PMC7872994; doi:10.1007/s00420-020-01570-1)
Supplement: Supplementary file 4 — Additional file4 (PDF 561 kb) [file 420_2020_1570_MOESM4_ESM.pdf]

Article title: The moderating role of psychosocial working conditions on the long-term relationship between depressive symptoms and work ability among employees from the Baby Boom generation
Journal name: International Archives of Occupational and Environmental Health
Author names: Jeannette Weber, Hans Martin Hasselhorn, Daniela Borchart, Peter Angerer, Andreas Müller
Corresponding author: Jeannette Weber, Institute of Occupational, Social and Environmental Medicine, Centre for Health and Society, Heinrich-Heine-University of Düsseldorf, Düsseldorf, Germany (email: jeannette.weber@uni-duesseldorf.de)

Online Resource 4

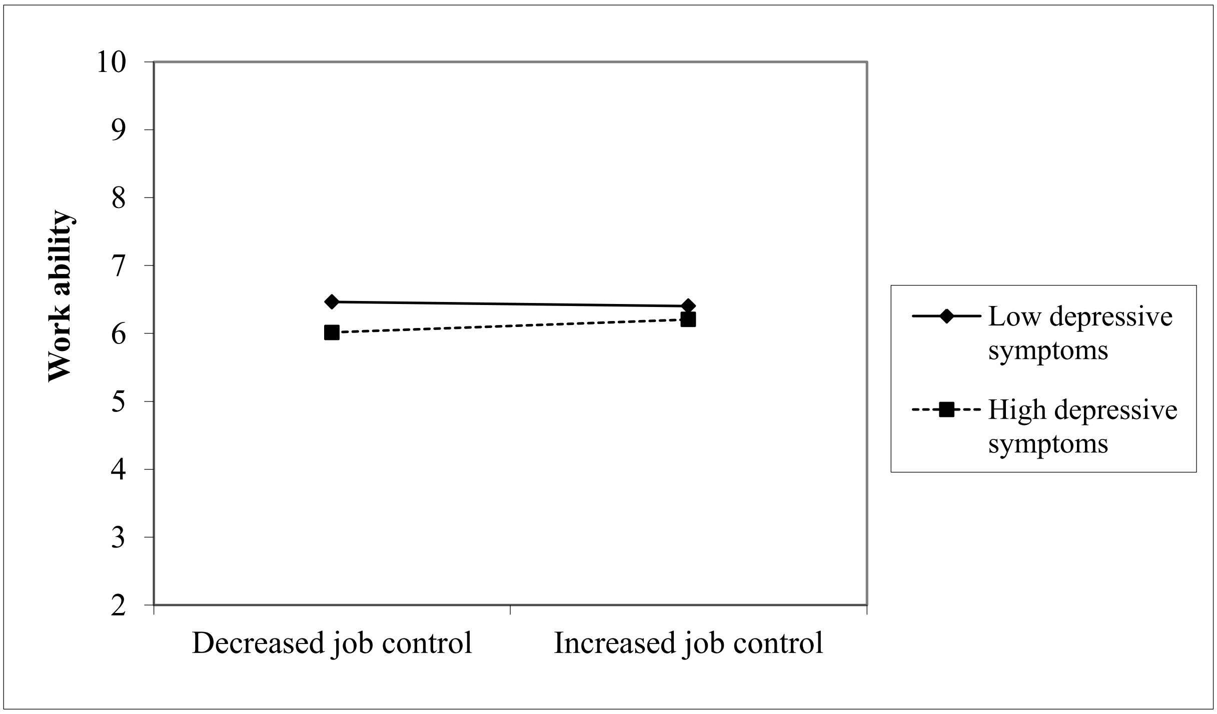

Interaction effect between depression and change in job control in male participants
