# Supplementary material for: The moderating role of psychosocial working conditions on the long-term relationship between depressive symptoms and work ability among employees from the Baby Boom generation
Source: Int Arch Occup Environ Health. 2020 Sep 8;94(2):295–307. doi: 10.1007/s00420-020-01570-1 (PMC7872994; doi:10.1007/s00420-020-01570-1)
Supplement: Supplementary file 5 — Additional file5 (PDF 580 kb) [file 420_2020_1570_MOESM5_ESM.pdf]

Article title: The moderating role of psychosocial working conditions on the long-term relationship between depressive symptoms and work ability among employees from the Baby Boom generation
Journal name: International Archives of Occupational and Environmental Health
Author names: Jeannette Weber, Hans Martin Hasselhorn, Daniela Borchart, Peter Angerer, Andreas Müller
Corresponding author: Jeannette Weber, Institute of Occupational, Social and Environmental Medicine, Centre for Health and Society, Heinrich-Heine-University of Düsseldorf, Düsseldorf, Germany (email: jeannette.weber@uni-duesseldorf.de)

Online Resource 5

Multiple linear regression analysis predicting work ability at wave 2, stratified for occupational group, data shown after imputation

	Unqualified work, n=1236		Qualified work, n=1613		Highly qualified work, n=728	
	B	95% CI	B	95% CI	B	95% CI
T1 Work ability	0.411*	0.360; 0.461	0.367*	0.322; 0.415	0.425*	0.356; 0.493
Sex (Ref. female)	-0.037	0.187; 0.113	0.072	-0.070; 0.215	0.258*	0.067; 0.450
Year of birth (Ref. 1959)	0.023	0.000; 0.047	0.033*	0.013; 0.054	0.013	-0.016; 0.043
Physical activity	0.087	-0.001; 0.175	0.129*	0.050; 0.207	0.035	-0.072; 0.143
Working conditions						
T1 Quantitative demands	-0.179*	-0.269; -0.090	-0.217*	-0.291; -0.143	-0.200*	-0.381; -0.081
T1 Control	0.019	-0.073; 0.111	0.103*	0.026; 0.180	-0.004	-0.120; 0.112
T1 Development	0.126*	0.036; 0.216	0.105*	0.010; 0.200	0.126	-0.020; 0.272
T1 Social Support	0.018	-0.067; 0.102	-0.027	-0.104; 0.051	0.115	-0.005; 0.235
T1 Leadership	0.101*	-0.010; 0.191	0.161*	0.080; 0.242	0.123*	0.007; 0.239
Symptoms						
T1 Depression	-0.258*	-0.335; -0.182	-0.142*	-0.212; -0.073	-0.133*	-0.242; -0.024
Change in working conditions						
Δ Quantitative demands	-0.255*	-0.333; -0.176	-0.320*	-0.395; -0.245	-0.304	-0.407; -0.201
Δ Control	-0.043	-0.119; 0.034	0.136*	0.060; 0.213	-0.002	-0.114; 0.111
Δ Development	0.159*	0.081; 0.238	0.171*	0.090; 0.253	0.202*	0.079; 0.326
Δ Social Support	0.110*	0.029; 0.192	-0.013	-0.087; 0.062	0.122*	0.008; 0.236
Δ Leadership	0.183*	0.098; 0.267	0.255*	0.180; 0.330	0.298*	0.179; 0.416
Interactions						
T1 Dep. * Δ Quantitative demands	-0.012	-0.072; 0.048	-0.011	-0.077; 0.055	0.048	-0.140; 0.044
T1 Dep. * Δ Control	-0.024	-0.085; 0.037	0.022	-0.045; 0.089	0.004	-0.113; 0.120
T1 Dep. * Δ Development	0.046#	-0.009; 0.100	-0.031	-0.098; 0.035	-0.024	-0.133; 0.086
T1 Dep. * Δ Social Support	-0.008	-0.071; 0.056	-0.010	-0.071; 0.050	0.026	-0.073; 0.124
T1 Dep. * Δ Leadership	-0.014	-0.078; 0.049	0.027	-0.036; 0.089	-0.026	-0.144; 0.044
Model fit: R^2	F(20,1215)=33.137*, R^2=.353		F(20,1592)=40.347*, R^2=.336		F(20,707)=20.355*, R^2=.365	

Note: T1 = wave 1, Δ = change from wave 1 to wave 2 with positive values representing an increase and negative values representing a decrease in working condition, n = number of participants, B = unstandardized regression coefficient, CI = confidence interval, Ref. = reference category; Levels of significance (two-tailed): * $p < 0.05$
